# Microsatellite mapping of QTL affecting growth, feed consumption, egg production, tonic immobility and body temperature of Japanese quail

**DOI:** 10.1186/1471-2164-6-87

**Published:** 2005-06-08

**Authors:** Francis Minvielle, Boniface B Kayang, Miho Inoue-Murayama, Mitsuru Miwa, Alain Vignal, David Gourichon, André Neau, Jean-Louis Monvoisin, Shin'ichi Ito

**Affiliations:** 1 Génétique et Diversité Animales, Institut National de la Recherche Agronomique, Centre de Jouy, 78352 Jouy-en-Josas, France; 2 Faculty of Applied Biological Science, Gifu University, 501-1193 Gifu, Japan; 3 Génétique Cellulaire, Institut National de la Recherche Agronomique, Centre de Toulouse, 31326 Castanet-Tolosan, France; 4 Unité Expérimentale de Génétique Avicole, Institut National de la Recherche Agronomique, Centre de Tours, 37380 Nouzilly, France; 5 Département de Génétique Animale, Institut National de la Recherche Agronomique, Centre de Jouy, 78352 Jouy-en-Josas, France

## Abstract

**Background:**

The Japanese quail (*Coturnix japonica*) is both an animal model in biology and a commercial bird for egg and meat production. Modern research developments with this bird, however, have been slowed down by the limited information that is available on the genetics of the Japanese quail. Recently, quail genetic maps with microsatellites and AFLP have been produced which open the way to comparative works with the chicken (*Gallus gallus*), and to QTL detection for a variety of traits. The purpose of this work was to detect for the first time QTL for commercial traits and for more basic characters in an F2 experiment with 434 female quail, and to compare the nature and the position of the detected QTL with those from the first chicken genome scans carried out during the last few years.

**Results:**

Genome-wide significant or suggestive QTL were found for clutch length, body weight and feed intake on CJA01, age at first egg and egg number on CJA06, and eggshell weight and residual feed intake on CJA20, with possible pleiotropy for the QTL affecting body weight and feed intake, and egg number and age at first egg. A suggestive QTL was found for tonic immobility on CJA01, and chromosome-wide significant QTL for body temperature were detected on CJA01 and CJA03. Other chromosome-wide significant QTL were found on CJA02, CJA05, CJA09 and CJA14. Parent-of-origin effects were found for QTL for body weight and feed intake on CJA01.

**Conclusion:**

Despite its limited length, the first quail microsatellite map was useful to detect new QTL for rarely reported traits, like residual feed intake, and to help establish some correspondence between the QTL for feed intake, body weight and tonic immobility detected in the present work and those reported on GGA01 in the chicken. Further comparative work is now possible in order to better estimate and understand the genetic similarities and differences of these two Phasianidae species.

## Background

Like the chicken, the Japanese quail belongs to the Phasianidae family [1]. In the wild, it is a small migratory bird originating from the Far East which was first raised in bird cages in Japan and China around the 15^th ^century because of its singing abilities, and later became to be known in Japan as a good egg-laying bird for human consumption [1]. Exported to North America in the 1930s, it started to be used for pilot studies in genetics and physiology and in avian research [2]. The Japanese quail is now a well established animal model in biology and a bird used for intensive egg and meat production mainly in Asia and Europe, but also in the Middle East and America [3].

Until recently, however, only a few linkage groups have been known for the Japanese quail, and few genes have been mapped [4] despite the large variety of traits that have been studied in the Japanese quail [3]. The development of the first microsatellite [5] and AFLP [6] panels and of the corresponding maps [6,7] have made it possible to localize new genes [8,9], which should further enhance the interest of the quail in biological research and open the way to more comparative genetic studies with chickens. Maps can also be used to do a genome-wide search for quantitative trait loci (QTL) by locating QTL nearby anonymous AFLP or microsatellite markers in segregating backcross or F2 populations. Moreover, the existence of markers that cross-amplify quail and chicken DNA [5,8] makes it possible to connect both maps and to compare the QTL results for these two species, thereby getting further insight into their evolutionary relationship.

The objective of the present work was to produce the first set of QTL detected in Japanese quail for growth, egg production and quality, feed consumption and other traits. For that purpose, an F2 experiment was carried out with two quail lines selected for early egg production [10] and for high duration of tonic immobility [11]. The microsatellite map [7] used for the detection of QTL in the present work was developed earlier from the same F2 individuals.

## Results

### Overall performances

Table 1 shows the number of observations, the mean, the standard deviation and the range of values for the traits recorded on the 434 F2 females of the experiment.

**Table 1:** Elementary statistics on the characters measured for the 434 F2 female Japanese quail of the genome scan

Trait^1^	N	Mean	Standard deviation	Minimum	Maximum
TI (s)	434	177.8	86.3	17.0	300.0
BT (C)	434	41.67	0.36	40.70	43.20
BW1 (g)	434	189.0	16.8	146.6	238.8
FI (g/d)	321	27.4	2.8	18.9	35.8
RFI (g/d)	321	0.0	1.8	-4.2	7.5
EW (g)	323	11.9	1.1	9.1	15.3
YW (g)	323	3.0	0.4	2.1	5.2
SW (g)	323	0.88	0.09	0.66	1.25
Y/A (%)	323	41	5	31	78
AFE (d)	429	44.2	5.2	37	87
EN	363	322.4	72.8	18	415
CL (d)	363	7.9	4.1	1.3	47.9
BW2 (g)	352	271.0	30.4	185.0	361.0

^1^: TI = tonic immobility at 8–9 days of age; BT = rectal temperature at 5 weeks of age; BW1 = 5-wk body weight; FI = feed intake at 30 weeks of age; RFI = residual feed intake at 30 weeks of age; EW = egg weight at 30 weeks of age; YW = yolk weight; SW = eggshell weight; Y/A = ratio of yolk weight over albumen weight; AFE = age at first egg; EN = number of eggs laid until the age of 69 weeks; CL = clutch length; BW2 = 70-wk body weight.

### QTL analysis

Table 2 shows the location of the significant QTL, their position on the chromosome, the maximum F value obtained at this position, their genetic effects, the reduction of the residual variance obtained by fitting a QTL at this location, and the corresponding chromosome-wide and genome-wide significance levels.

**Table 2:** Chromosomal location, test statistic (F), genetic effects and significance of the QTL detected in the Japanese quail

Chromosome (map length cM)	Trait^1^	Position (cM)	Flanking markers	F	Additive effect ± SE	Dominance effect ± SE	Reduction of σ^2 ^(%)	Chromosome-Wide Probability	Genome-Wide	
									
									Probability	Level
CJA01 (206)	CL	0	*GUJ0055*	9.9	-0.93 ± 0.36	-1.81 ± 0.58	4.9	0.0006	0.002	very significant
	BW2	18	*GUJ0055-GUJ0052*	11.6	-1.29 ± 0.27	ns^2^	5.7	0.0002	0.0005	very significant
	BW1	19	*GUJ0052*	5.9	-4.36 ± 1.38	ns	2.3	0.034	0.09	suggestive
	FI	19	*GUJ0052*	8.3	-1.03 ± 0.26	ns	4.4	0.004	0.01	significant
	TI	91	*ADL0037*	6.2	-20.3 ± 6.3	ns	2.5	0.034	0.09	suggestive
	BT	180	*GUJ0062*	5.7	-0.088 ± 0.026	ns	2.5	0.047	0.12	ns
	SW	191	*GUJ0062-GUJ0068*	7.2	-0.027 ± 0.009	-0.030 ± 0.0134	3.4	0.016	0.04	significant

CJA02 (61)	BW1	54	*GUJ0063-GUJ0027*	4.6	ns	7.42 ± 2.75	1.7	0.038	0.29	ns

CJA03 (38)	BT	1	*GUJ0099*	6.1	-0.090 ± 0.029	ns	2.5	0.008	0.11	ns

CJA05 (21)	SW	12	*GUJ0049*	5.5	-0.026 ± 0.008	ns	2.3	0.012	0.27	ns

CJA06 (74)	EW	0	*GUJ0021*	5.4	0.330 ± 0.100	ns	2.7	0.023	0.16	ns
	EN	32	*GUJ0087*	7.0	21.6 ± 6.1	ns	3.3	0.006	0.04	significant
	AFE	34	*GUJ0087-GUJ0054*	6.2	-1.52 ± 0.46	ns	2.9	0.012	0.09	suggestive

CJA09 (25)	BW1	25	*GUJ0071*	4.3	3.34 ± 1.22	ns	1.6	0.028	0.46	ns

CJA14 (8)	BW2	7	*GUJ0023-GUJ0097*	6.2	ns	-1.32 ± 0.38	2.9	0.004	0.24	ns

CJA20 (25)	FI	2	*GUJ0065-GUJ0083*	8.5	-0.883 ± 0.231	ns	4.5	0.001	0.02	significant
	SW	21	*GUJ0065-GUJ0083*	6.7	-0.026 ± 0.008	0.024 ± 0.013	3.4	0.003	0.06	suggestive
	RFI	25	*GUJ0083*	7.2	-0.525 ± 0.142	ns	3.8	0.002	0.04	significant

^1^: CL = clutch length (d); BW2 = 70-wk body weight (g); BW1 = 5-wk body weight (g); FI = feed intake at 30 weeks of age (g/d); TI = tonic immobility at 8–9 days of age (s); BT = rectal temperature at 5 weeks of age(C); SW = eggshell weight at 30 weeks of age (g); EW = egg weight (g); EN = egg number until the age of 69 weeks; AFE = age at first egg (d); RFI = residual feed intake at 30 weeks of age (g/d).

^2^: ns = not significant.

Out of the 18 QTL found to be chromosome-wide significant (p_c _< 0.05) on 8 of the 12 autosomes scanned in this work, 7 were genome-wide significant (p_g _< 0.05), and 4 were genome-wide suggestive (p_g _< 0.10). Genome-wide significant QTL for early (BW1) and late (BW2) measures of body weight were only found on CJA01, but there was a chromosome-wide significant QTL for BW1 on CJA02 and on CJA09, and for BW2 on CJA14. Significant QTL were found for feed intake (FI) on CJA01 and for FI and residual feed intake (RFI) on CJA20. Genome-wide significant or suggestive QTL for egg production characters were obtained, on CJA01 for clutch length (CL), and on CJA06 for total egg number (EN) and age at first egg (AFE). There were QTL for eggshell weight (SW) on CJA01 and CJA20, and some evidence for another one on CJA05. One genome-wide suggestive QTL was found for tonic immobility (TI) on CJA01, and there was marginal evidence of QTL for egg weight (EW) on CJA06, and for body temperature (BT) on CJA01 and CJA03.

In most cases, the genetic effect was additive and negative, indicating that the QTL allele with the lowest additive effect originated from Line DD. The few exceptions were for QTL on CJA06 and CJA09. Among genome-wide suggestive and significant QTL, dominance was only significant for CL and SW. The reduction of the residual variance due to fitting a QTL was moderate and did not exceed 5.7%.

### Chromosome CJA01

**Figure 1:**
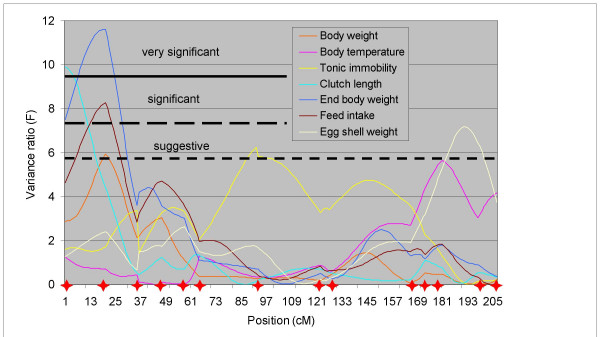
**Interval mapping of QTL on chromosome CJA01. **Only traits with a QTL detected on CJA01 are shown. Significance thresholds varied little between traits, so unique genome-wide suggestive (p < 0.10), significant (p < 0.05) and very significant (p < 0.01) thresholds are indicated by horizontal dashed or continuous lines. The positions of the markers are indicated by red stars.

Interval mapping results for CJA01 are shown in Table 2 and Figure 1. For all traits, the allele received from the Line DD ancestry decreased the additive genotypic value. The QTL for BW1, BW2 and FI were found at very close positions, the QTL for TI was obtained in the middle of the interval near the chicken microsatellite *ADL0037*, and the other QTL were placed at both ends of the chromosome.

### Chromosome CJA06

**Figure 2:**
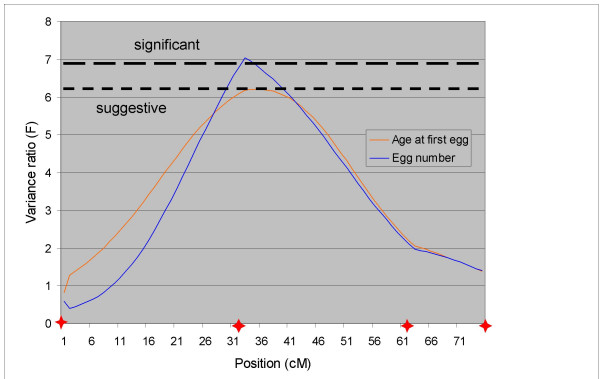
**Interval mapping of QTL on chromosome CJA06. **Only traits with a QTL detected on CJA06 are shown. Significance thresholds varied little between traits, so unique genome-wide suggestive (p < 0.10) and significant (p < 0.05) thresholds are indicated by horizontal dashed lines. The positions of the markers are indicated by red stars.

Interval mapping results for CJA06 are shown in Table 2 and Figure 2. The QTL for EN and AFE were found in the middle of the chromosome at very close positions. Both alleles received from the Line DD were favourable for egg production, with a positive additive effect on EN and a negative one on AFE.

### Chromosome CJA20

Interval mapping results for CJA20 are shown in Table 2. In this short linkage group, the QTL for FI and RFI were found at opposite ends but, for both traits, the allele decreasing the additive value was transmitted by the Line DD.

### Imprinting and two-QTL alternative

Imprinting (parent-of-origin effect) was estimated for genome-wide significant QTL. It was only found to be significant for BW1 (F_s _= 9.0 > F_.01 [1,415] _= 6.7) and FI (F_s _= 7.6 > F_.01 [1,318] _= 6.7), on CJA01, with a larger effect (5.7 ± 1.4 g and 0.73 ± 0.25 g, respectively) for sire-transmitted alleles. Similarly, the two-QTL hypothesis was tested, but there was no evidence for two different QTL located on the same linkage group and acting simultaneously on the same trait.

## Discussion

### Method

The line-cross model used for QTL detection assumes that different QTL were fixed in the Lines DD and LTI [12]. This hypothesis could not be checked, but bandsharing (BS) between the two quail origins was low (0.19) [13] and similar to that between highly divergent poultry lines of broilers and layers [14], suggesting that the departure from the ideal situation of fixation in the parental lines was limited. Moreover, the main consequence of starting from outbred F0 lines would only be to underestimate the QTL effects [15]. This might partly explain the relatively small reduction of residual variance observed (see Table 2) for significant QTL under the full model. Using a more general approach with full/half sib models [16] was also possible, but it would have required larger full-sib families [17] in order to accurately estimate the larger number of parameters needed with those models.

### QTL effects

The additive effects listed in Table 2 estimated the differences between Line DD and Line LTI alleles. For body weight and feed consumption QTL, the negative sign of the additive effects indicated that favourable alleles (increasing body weights and decreasing FI and RFI) originated from Line DD, in agreement with the results of the zootechnical comparison between the two lines at the F0 generation of the present experiment [18]. The situation was similar for the QTL found for EN (positive difference) AFE (negative difference) and SW (negative difference), since Line DD was a better laying line but with lower egg (and eggshell) weight. Surprisingly, however, the best allele for the very significant QTL found for CL was transmitted by Line LTI, although its average clutch length was 1.1 d shorter than that of Line DD in the F0 generation [18]. Of course, an inferior line may possess superior alleles as first evidenced in early tomato QTL studies [19]. Also, this QTL had a larger dominance effect (see Table 2), which could not be forecasted from information on the pure lines. It is also possible, however, that its localisation at one end of the linkage group and its effect were some artefact related to the significant segregation distortion observed at this position. The QTL allele originating from the LTI line selected for extreme duration of tonic immobility [11] also increased TI in the F2 population. Its position on CJA01 confirmed the result obtained for TI in another F2 experiment on the Japanese quail carried out to detect stress-related traits using AFLP markers [9]. To our knowledge, the location of a significant QTL for residual feed intake in layers is the first one in a poultry species. The QTL was located on a microchromosome with few markers, however, and it will need to be confirmed in independent genome scans, but it is already an interesting starting point for further work. Finally, it was not surprising that the QTL for BT were only chromosome-wide significant in this medium-sized experiment because body temperature is highly regulated and shows very little variation (see Table 1), but quantitative genetic variation for BT has already been reported in the chicken [20], and confirmation of these QTL would represent a very significant finding.

### Co-location of QTL

It is likely that the QTL for BW2, BW1 and FI, located at 18 and 19 cM on CJA01, represent a single gene. Indeed, body weight and feed intake are known to be highly correlated genetically (0.5 to 0.9) in poultry [21], and the rank correlation between BW1 and FI was 0.5 (p < 0.01) in the F2. It is worth noting, however, that the suggestive QTL found for TI on the same chromosome was not placed near any other significant QTL. This observation was in good agreement with the absence of association between TI and all the other traits reported from factorial correspondence analysis of the same F2 data [22]. Therefore, the association between stress susceptibility and production in the quail, if it exists, is not mediated significantly by major genes involved in the determinism of the duration of tonic immobility measured at a young age.

The only other instance of close location of two QTL was for EN and AFE at 32 and 34 cM on CJA06, but the rank correlation between them was only -0.1 (p < 0.05) in the F2, which does not strongly suggest that there might be also a single pleiotropic QTL at this position.

Unfortunately, pleiotropy could not be tested with the software presently available.

### Comparison with chicken QTL

Several recent reports of genome-wide scans in the chicken have described QTL for body weight [23-30], feed intake [23,25,27], egg production traits [25,27,28] and tonic immobility [31], but none was found for body temperature and residual feed intake in layers. A QTL was found for RFI in broilers on GGA01, GGA04 and GGA05, however, but in studies [32,33] which were focused on some specific chromosomal regions.

Significant QTL for body weight in the chicken were only reported repeatedly on GGA01 [23,24,26,27,30], homologous to CJA01, and on GGA04 [25,28,32]. Moreover, in two out of the three chicken studies where both BW and FI were measured, QTL were found at the same position for body weight and feed intake on GGA01 [23,27], as on CJA01 in the present work. In the third report [25], despite the varied nature of the traits which were studied, no QTL was found at all on GGA01, but QTL which affected BW and TI were detected in the same region of GGA04. In our work, however, no QTL was found on CJA04. Overall, then, the largest chicken and quail chromosomes might contain an important and homologous QTL for growth. Regarding QTL for egg production in layers, none were found on GGA06 which is homologous to CJA06 on which QTL for EN and AFE have been detected in this work. But egg production is a complex trait because egg laying evolves with time, and it was only measured over a limited period in the works on the chicken, so the characters were not directly comparable.

The location of a QTL for tonic immobility on two homologous chromosomes in chicken [31] and quail suggests that the duration of tonic immobility, a trait associated to fear response [11], is, at least partly, under a similar genetic determinism in the two species. The associations found between QTL for TI and QTL for growth and egg weight on GGA01 in the red jungle fowl layer intercross [31], however, contradict the lack of association reported in our quail QTL experiment in the present paper, and reported in previous phenotypic analyses [18,22].

Some parent-of-origin effects were recently reported in the chicken [34]. They were detected mainly on GGA01, and some were found for QTL for body weight and feed intake, as in the present study. It is rather unlikely that these converging results obtained independently in two species from the family Phasianidae were only coincidental. Consequently, they should raise some interest in looking further for this type of effect in avian species (where reciprocal effects are common), and, more generally, for possible imprinting-like mechanisms in Birds [34].

### Possible candidate genes

Of course, only chromosomal regions could be detected in this study, but it is interesting to note that in the chicken, the gene for the insulin-like growth factor IA associated with growth [35], and the gene for a receptor of serotonin which might be implicated in behaviour [36] are on GGA01 , whereas we found a QTL for body weight and one for tonic immobility on the homologous quail chromosome CJA01. In the same way, the agouti gene is linked to feed intake [37], and a QTL for feed intake was detected on CJA20 where an agouti-like locus was mapped in the Japanese quail [8]. These chromosomal co-locations are only indicative, but they might deserve to be explored further.

## Conclusion

For the first time with the Japanese quail, a partial genome scan using microsatellites has revealed genome-wide significant QTL for major production-related traits (BW, FI, CL, EN), and QTL for other traits with wider potential interest (TI, RFI, BT) were also detected. Few results for traits measured on both the quail and chicken are available, and comparisons between the two species are only at the beginning, but the common co-location (CJA01 and GGA01) of QTL acting on BW and FI, and of QTL for TI are already indicative of the detailed genetic similarity between the two species that future comparative work should further explore. Of course, only chromosomal regions could be detected in this study, but it is interesting to note that in the chicken, the gene for the insulin-like growth factor associated with growth and the gene for the serotonin 1F receptor implicated in behavioral traits are on GGA01, whereas a QTL for body weight and one for tonic immobility were found on CJA01. In the same way, the agouti gene is linked to feed intake, and a QTL for feed intake was detected on CJA20 where an agouti-like locus was just mapped in the Japanese quail.

## Methods

### Birds and Husbandry

Two lines, LTI and DD, with different origins [13] and selected respectively for high duration of tonic immobility [11] and for early egg production [10] were crossed reciprocally (12 single-pair matings) to produce F1 quail. All LTI breeders had a maximum duration (300 s) of experimental tonic immobility, and a maximum value for TI was 55 s in Line DD. Ten males and 30 egg-laying females were drawn randomly across F1 families to produce the F2 generation. The duration of TI in the F1 breeding group varied between 31 and 300 s. Each F1 sire was mated to three full sisters from another F1 family, and 30 F2 full-sib families were produced in three consecutive hatches to obtain the 434 female quail studied in the present work, with 39 to 45 F2 birds per sire family and 12 to 19 quail per full-sib F2 family.

F2 quail were successively given standard starter and commercial layer diets [10]. At 5 wk of age they were assigned at random to individual cages of a 4-tier battery maintained at 25°C, and in which they remained until the end of the experiment, with free access to feed and drinking water.

### Traits

The traits will be described chronologically. The duration of TI was measured for up to 5 min at 8 or 9 days of age. TI was the length of time during which a chick remained immobile after the freezing reaction had been induced by keeping it gently on its back for 10 s. Body weight (BW1) and rectal body temperature (BT) were measured at 5 weeks of age. Egg production was recorded daily and individually from 5 to 69 weeks of age. Age at first egg (AFE), total egg number (EN) and clutch length (CL: average number of consecutive days with an egg) were obtained from the egg laying data. A 24 day feed trial was conducted on 30-wk females fed ad libitum. Individual daily intake was recorded, and total residual feed intake was estimated for each bird as the residual of the multiple regression of total feed intake on mean metabolic body weight, body weight gain and egg mass produced on test [22]. Observed and residual intake values were then divided by 24 to obtain FI and RFI. RFI is a relative value which represents the daily amount of feed used for non-productive traits (activity, basal metabolism, heat increment). It is an important character for production purposes but also for more fundamental metabolic studies. Egg weight (EW) and gross composition (YW: yolk weight, SW: eggshell weight; Y/A: ratio of yolk weight over albumen weight) were obtained from three consecutive eggs per quail collected around 30 weeks of age for each quail. Finally, body weight was measured again at 70 weeks of age (BW2). Statistical analyses were run with untransformed data for all characters, except for tonic immobility which was also analysed as log(1+TI), and produced the same results as for TI.

### Genotyping

All 24 F0, 40 F1 and 434 F2 quail in the present experiment belonged to the resource family set up to establish the first microsatellite quail map [7], and they had all been typed previously for the microsatellites listed in the augmented microsatellite panel [5]. Genotyping was carried out at the Gifu University, and genotypic data were arranged, validated and stored in the MAPGENA database. Mean heterozygosity of the 10 F1 sires was 61%. The map had been built from 72 microsatellite loci, and 58 of them had been resolved into 13 linkage groups, including a Z chromosome group, for a total map distance of 576 cM with a 10 cM average spacing between loci [7]. At that time, only seven of the linkage groups could be assigned to chromosomes CJA01, CJA02, CJA05, CJA06, CJA14, CJA27 and CJAZ through comparative mapping with the chicken. Since then, however, all other linkage groups in the microsatellite map were assigned to other chromosomes [Kayang *et al*., unpublished data; [8]]. Consequently, the remaining linkage groups Q03, Q04, Q08, Q09, Q10 and Q11 in the original article [7] have been renamed respectively, CJA03, CJA13, CJA09, CJA04, CJA20 and CJA10 in the present one.

### QTL analyses

QTL detection was performed using the line cross method [12] implemented in the QTL Express software [38]. Briefly, for each trait independently, an analysis of regression was carried out along each autosome to test for the effect of a putative QTL. In the present study, the linear model also included a hatch effect (3 classes) for all traits, and a technician effect (2 classes) for TI, so an analysis of covariance was carried out. The most likely position of the QTL corresponded to that found with the maximum F value. Five thousand permutations [39] were then carried out to set significance levels (p_c_) for the most likely chromosome-wide QTL. Finally, genome-wide significance (p_g_) for a QTL detected on a given chromosome was obtained from p_c _by: p_g _= 1-(1-p_c_)^1/r^, where r was the ratio of the length of this chromosome over the total chromosome length (545 cM) spanned by the present study. Chromosome-wide significance was set up conservatively at p_c _= 0.05. Genome-wide significant and suggestive thresholds were set up respectively at p_g _= 0.05 and 0.10. A significant (p < 0.05) segregation distortion was found only near the 0 position on CJA01.

## Authors' contributions

FM designed and coordinated the study, and wrote the paper. BBK, SI, MIM and MM designed, organized, and conducted all the microsatellite work. AV led the mapping part. AN was responsible for the data bank, and DG and JLM supervised and carried out the data collection.
